# Molecular Strategies to Target Protein Aggregation in Huntington’s Disease

**DOI:** 10.3389/fmolb.2021.769184

**Published:** 2021-11-12

**Authors:** Olga D. Jarosińska, Stefan G. D. Rüdiger

**Affiliations:** ^1^ Cellular Protein Chemistry, Bijvoet Centre for Biomolecular Research, Utrecht University, Utrecht, Netherlands; ^2^ Science for Life, Utrecht University, Utrecht, Netherlands

**Keywords:** proteostasis, huntingtin (HTT), protein fibrils, aggregation, protein quality control, protein degradation, huntington’s disease, mRNA degradation

## Abstract

Huntington’s disease (HD) is a neurodegenerative disorder caused by the aggregation of the mutant huntingtin (mHTT) protein in nerve cells. mHTT self-aggregates to form soluble oligomers and insoluble fibrils, which interfere in a number of key cellular functions. This leads to cell quiescence and ultimately cell death. There are currently still no treatments available for HD, but approaches targeting the HTT levels offer systematic, mechanism-driven routes towards curing HD and other neurodegenerative diseases. This review summarizes the current state of knowledge of the mRNA targeting approaches such as antisense oligonucleotides and RNAi system; and the novel methods targeting mHTT and aggregates for degradation via the ubiquitin proteasome or the autophagy-lysosomal systems. These methods include the proteolysis-targeting chimera, Trim-Away, autophagosome-tethering compound, autophagy-targeting chimera, lysosome-targeting chimera and approach targeting mHTT for chaperone-mediated autophagy. These molecular strategies provide a knowledge-based approach to target HD and other neurodegenerative diseases at the origin.

## 1 Introduction

### 1.1 Huntington’s Disease

**FIGURE 1:**
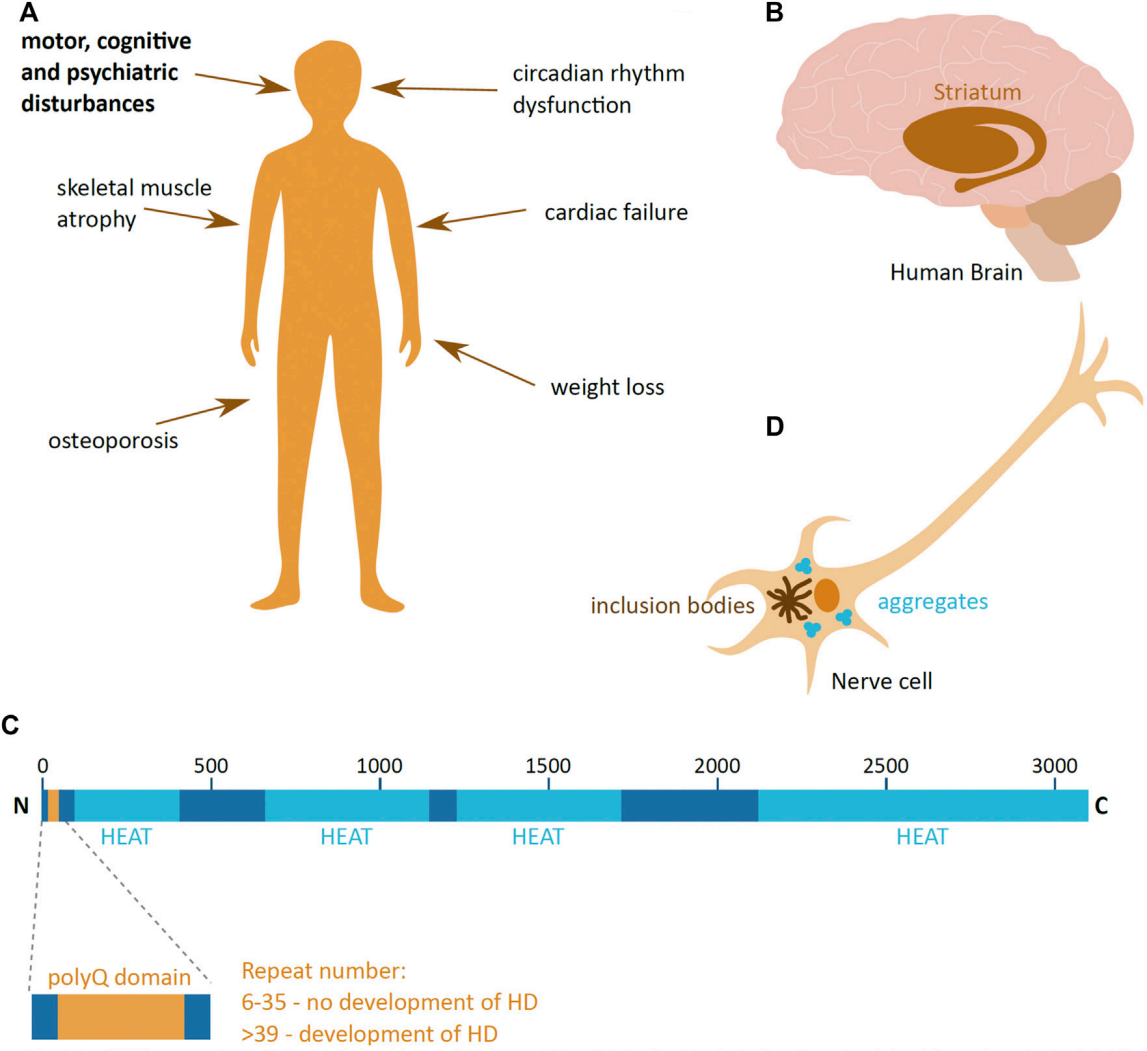
Huntington’s disease. **(A)** Symptoms of Huntington’s disease (HD); the main triad of symptoms is highlighted in bold. **(B)** The part of the human brain predominantly affected in HD (brown), the striatum. **(C)** Schematic presentation of the domain organisation of huntingtin (HTT) protein, with the polyglutamine region (PolyQ) (yellow) and HEAT domains (light blue). The repeat numbers indicate the risk of developing HD. Image adapted from Guo et al., 2018. N and C indicate the protein N- and C-terminus; numbers indicate the amino acid length of HTT. **(D)** Schematic presentation of a HD hallmark—aggregates and inclusion bodies in the cytoplasm of a nerve cell.

Huntington’s disease (HD) is a neurodegenerative disease, caused by the aggregation of the huntingtin (HTT) protein in the human brain nerve cells. It is a rare disease with the incidence rate of 5–10 per 100,000 in most of Europe, South and North America, and lower in Asia and Africa (Reiner et al., 2011). This autosomal dominant disease affects males and females at the same frequency; the onset of the disease is usually around 40 years old and death occurs typically 20 years after diagnosis. The disease manifests itself through a triad of disturbances in the motor, cognitive and psychiatric functions (Figure 1A). As the disease progresses over time, it has a profound effect on the quality of life, finally leading for the need of 24 days care of a patient (McColgan and Tabrizi, 2018).

HD leads to the loss of nerve cells and brain mass, and the most prominently affected are the neurons of the striatal part of the basal ganglia (McColgan and Tabrizi, 2018) (Figure 1B). As the disease progresses, neuronal loss and shrinkage spreads to other parts of the brain at varying degrees depending on the stage of the disease. Eventually, in advanced stages of HD, the loss of brain weight can be up to 25% (McColgan and Tabrizi, 2018). HD not only affects the brain, but also has a detrimental effect in other tissues, leading to weight loss, atrophy of skeletal muscles, cardiac failure, osteoporosis and dysfunction in the circadian rhythm (van der Burg et al., 2009; Smarr et al., 2019) (Figure 1A).

### 1.2 HTT Protein

Responsible for HD is a mutant form of the HTT protein. HTT is encoded by the *HTT* gene on chromosome 4, and is 3,142 amino acids long. It is a largely α-helical protein that comprises an N-terminal domain containing an extensive polyglutamine (polyQ) stretch, encoded by a repeat of CAG nucleotides, and multiple 50 amino acid-long HEAT (Huntingtin, Elongation factor 3, protein phosphatase 2A, the yeast kinase TOR1) domains (Guo et al., 2018) (Figure 1C). The HEAT domains act as scaffolds for protein-protein interaction (Ratovitski et al., 2012). The HTT protein is produced ubiquitously in the human body, and even though the precise function of HTT is not clear, it is suggested to be involved in transcription regulation (Zuccato et al., 2003), neuronal vesicle transport (Szebneyi et al., 2003), cell morphology and cytoskeleton function (Tousley et al., 2019) and the immune system (Regan et al., 2020). In addition, HTT importance changes during organism development, where it is vital during early animal development (Nasir et al., 1995), and its depletion in young and adult mice causes the development acute pancreatitis and alterations in brain homeostasis, respectively (Wang et al., 2016; Dietrich et al., 2017).

Disease-causing mutant HTT (mHTT) has an expanded polyQ domain, which is caused by the increase in number of glutamine encoding CAG repeats. The number of repeats in healthy individuals ranges from 6 to 35, and individuals who have more than 39 repeats of CAG are certain to develop HD (McColgan & Tabrizi, 2018) (Figure 1C). The number of repeats negatively correlates with HD age-of-onset, and very high numbers of repeats can cause juvenile onset HD, which has a more rapid progression rate than adult onset HD. This polyQ expansion mutation induces a dominant and cumulative toxic gain-of-function mutation of the protein, which is highly prone to aggregation. The pathophysiological hallmark of HD is the formation of mHTT aggregates and inclusion bodies inside cells (Turmaine et al., 1997) (Figure 1D), which lead to cell quiescence and ultimately to cell death (Reiner et al., 2011).

### 1.3 Treatment

Whereas HD is a neurodegenerative disease, with a known monogenic etiology and well identified disease-causing agents, there is still no treatment. The advances in the understanding of the molecular mechanisms of HD allow for new molecular strategies for drug development, by targeting HTT protein aggregation. These strategies mainly focus on lowering the levels of mHTT in the cells (Evers et al., 2018; Imbert et al., 2019; Li et al., 2019; Spronck et al., 2019; Caron et al., 2020).

This review describes the current state of knowledge of the molecular strategies that can target protein aggregation in HD. First, it describes the molecular mechanisms of HD and the potential targets for HD treatment, and then summarizes the different molecular strategies researched to target mHTT protein aggregation. These strategies work by either targeting the mHTT mRNA or protein for degradation.

## 2 Main Body

### 2.1 Molecular Mechanisms of Huntington’s Disease

#### 2.1.1 Molecular Mechanism of polyQ Expansion

The polyQ extensions in mHTT is inherited in an autosomal dominant manner and leads to a gain-of-function, whereby the mutant induces a toxic effect regardless of the presence of the normal HTT. The number of repeats inherited leads to somatic instability of the region, which causes the expansion of the polyQ repeat domain over time, which in turn aggravates the severity of the mutation (Kennedy et al., 2003). Additionally, a number of single nucleotide polymorphisims (SNPs) are linked to the CAG expansion, suggesting its involvement in the mutation (Warby et al., 2009; Carroll et al., 2011).

Even though HTT is expressed ubiquitously in the body, mHTT has the biggest cytotoxic effect in nerve cells, predominantly in the striatum. This could be due to the differential transcription of HTT mRNA in cells of neuronal and non-neuronal origin, where nerve cells have more HTT mRNA present in the cell nucleus, suggesting higher protein levels favouring aggregation (Didiot et al., 2018). Secondly, nerve cells show to be more prone to CAG region instability leading to its expansion, compared to non-neuronal glia cells (Kennedy and Shelbourne, 2000; Shelbourne et al., 2007). The third possible reason is the different processes regulating protein aggregation in specific parts of the brain, which lead to the accumulation of mHTT aggregates only in the striatum and not in the other brain parts, as shown in aged mice (Yang et al., 2020).

#### 2.1.2 Pathogenic mHTT Protein Variant

The development of HD is driven by the expansion of the polyQ region of HTT. However it is not the whole protein that is the involved in the pathophysiology, but only the N-terminal fragments, predominantly the ones encoded by exon 1, are identified as the cytotoxic variants (Mangiarini et al., 1996; Barbaro et al., 2015). The toxic variants are referred to as mHTT. mHTT aggregates and gives rise to nuclear and cytoplasmic inclusions (Lunkes et al., 2002; Landles et al., 2010), and its overexpression in mice brains leads to age-dependent accumulation in the striatum leading to the HD-like phenotypes (Yang et al., 2020). The N-terminal fragments were identified in human HD brains post mortem (Lunkes et al., 2002).

The N-terminal fragment can be generated through aberrant splicing of mRNA or through protease-mediated cleavage of the protein, and both mechanisms are affected by the pathogenic expansion of the polyQ region. The expanded polyQ domain leads to the incomplete pre-mRNA splicing of exon 1 to exon 2 (Neueder et al., 2018). The resulting mRNA has a stop codon after the exon 1, which in turn leads to the translation of only the exon 1 fragment (Sathasivam et al., 2012). HTT protein cleavage by caspase and calpain proteases occurs both in healthy and in HD brains (Kim et al., 2001). However, cleavage at specific sites of mHTT leads to the generation of cytotoxic fragments and development of HD (Gafni et al., 2004; Graham et al., 2006). Additionally, a HD-specific SNP, which introduces a missense mutation, alters the post-translational modifications of the protein and can induce different cleavage of the protein, leading not only to generating the toxic mHtt forms, but also an aggregating form of wild-type HTT (wtHTT) (Martin et al., 2018).

**FIGURE 2:**
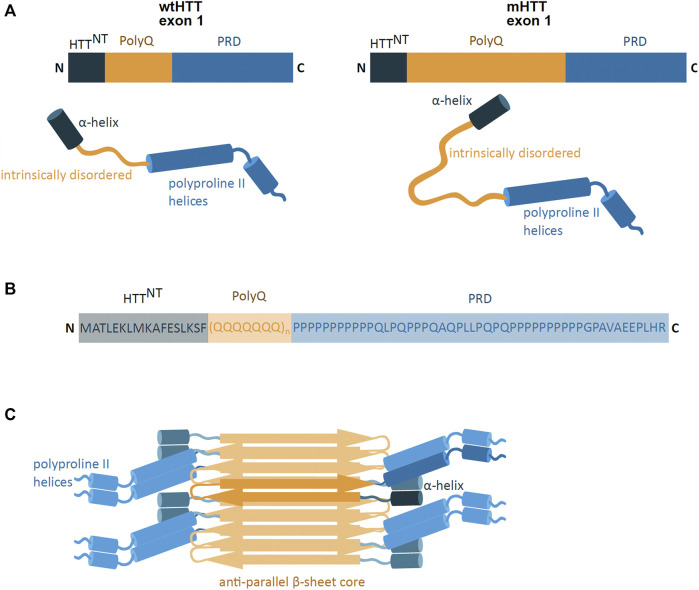
mHTT exon 1 structure and aggregation model. **(A)** Schematic presentation of primary and secondary structure of wild-type HTT (wtHTT) and mutant HTT (mHTT) exon 1, showing the HTT^NT^ (dark blue; α-helix), polyQ domain (yellow; intrinsically disordered) and proline-rich domain (PRD) (lighter blue; polyproline II helices). mHTT contains an expanded polyQ domain compared to wtHTT. N and C indicate the protein N- and C-terminus. **(B)** The amino acid sequence of the exon 1 mHTT protein. Highlighted are the identified regions of HTT^NT^, PolyQ and PRD. The protein sequence is represented by one letter amino acid code. **(C)** Model of mHTT exon 1 HTT fibril architecture: mHTT interacts through the anti-parallel β-sheet (orange), with flanking HTT^NT^ and PRD domains (blue) overhanging at the sides of the fibril; single monomer highlighted in darker colours. (adapted from Boatz et al., 2020).

The pathogenic exon 1 of the mHTT protein consists of three domains, including the polyQ sequence flanked by N- and C-terminal domains (Figures 2A,B). The polyQ domain is a conformationally versatile peptide which can take on different structures, such as intrinsically disordered, an α-helical conformation near the N-terminal domain, or a β-sheet confirmation found in mHTT aggregates (Boatz et al., 2020; Urbanek et al., 2020). These polyQ conformations are highly influenced by conformational features of the flanking N- and C-

Terminal regions (Urbanek et al., 2020). The N-terminal region (HTT^NT^) is a 17 amino acid long sequence and predominantly takes on the form of a compact α-helical coil structure (Thakur et al., 2009). The coiled HTT^NT^ acts as an anchor tethering the protein to intracellular membranes and its compact structure resists aggregation (Thakur et al., 2009; Tao et al., 2019). However, the polyQ sequence can influence the HTT^NT^ conformation, whereby the expansion of the polyQ region induces HTT^NT^ unfolding and promoting its self-aggregation (Thakur et al., 2009; Boatz et al., 2020). The C-terminus is a proline-rich domain (PRD), which adopts an intrinsically disordered confirmation with polyproline II helices (Boatz et al., 2020). In contrast to HTT^NT^, the PRD sequence has a suppressive effect on the polyQ protein aggregation potential (Bhattacharyya et al., 2006).

#### 2.1.3 Exon 1 Protein Aggregation

The formation of mHTT aggregates is self-initiated and is dependent on the polyQ region length, protein concentration and time (Boatz et al., 2020; Scherzinger et al., 1999). The mHTT protein is able to spontaneously aggregate and form different structures such as monomers, oligomers and fibril-rich inclusion bodies (IB) (Olshina et al., 2010; Wagner et al., 2018). The oligomeric aggregates are small, soluble, and are found in the nucleus and in the cytoplasm of the cell (Kim et al., 2016; Yang et al., 2020). The IB, on the other hand, are insoluble protein aggregates, found in both the cytoplasm and in the nucleus (Bäuerlein et al., 2017). IB are mostly spherical and made of fibrils (Bäuerlein et al., 2017), which are long rod-like structures of tightly packed mHTT proteins (Boatz et al., 2020). The mHTT fibrils are amyloid-like structures where the mHTT polyQ region forms a tightly packed, highly rigid and dehydrated anti-parallel β-sheet core flanked by mHTT N- and C-terminal domains (Boatz et al., 2020) (Figure 2C). The molecular architecture of the deposits share a number of features with aggregates found in Alzheimer’s and Parkinson’s disease (Boatz et al., 2020).

Notably, these structural data are derived from artificial systems. In other neurodegenerative diseases such as Alzheimer’s disease and Parkinson’s disease the shape of fibrils obtained from primary material differ in structure from fibrils produced *in vitro* (Scheres et al., 2020; Schweighauser et al., 2020). It remains to be seen how closely artificial HTT structures resemble fibrils *in vivo*.

The process of HTT aggregation has an initial a lag phase, followed by an exponential growth phase and then plateau. The aggregation of mHTT is initiated by primary nucleation where a spontaneous aggregation nucleus induces the conformation change of other proteins promoting their addition to the aggregate, through templated aggregation (Wagner et al., 2018). The size of the nucleus can range from a monomer to a oligomer (Chen et al., 2002; Thakur et al., 2009). The conformational changes of the N-terminal domain of mHTT, caused by the expansion of the polyQ region, accelerate nucleation and aggregate formation (Thakur et al., 2009; Boatz et al., 2020).

Aggregates expand through the interactions between the polyQ repeat regions, which adopt a β-sheet conformations (Boatz et al., 2020). These aggregates elongate into fibrils, speeding up the growth process, by recruiting new monomers. Elongating fibrils can induce fibril-dependent nucleation, leading to the formation of fibril branches (Wagner et al., 2018). Fibrillary branching is seen in HTT aggregates, but not in other amyloid fibrils (Boatz et al., 2020). Interestingly, the elongating fibrils can also recruit wtHTT (Chen et al., 2001; Slepko et al., 2006), which naturally do not form fibrillary structures.

#### 2.1.4 Effect of polyQ Extensions on Cellular Function

HTT oligomers and IBs greatly impact cell function by attracting and sequestering a number of proteins involved in processes of energy production, protein trafficking, RNA post-translational modifications and cell death (Ratovitski et al., 2012; Kim et al., 2016; Ramdzan et al., 2017). This altering of key cellular functions leads to the complete dysregulation of the cell, causing it to gradually slow-down and ultimately resulting in cell death.

In cell culture models, mammalian soluble mHTT oligomers can interact with up to 800 different proteins (Kim et al., 2016). The protein interaction is dependent on the length of the polyQ repeat of mHTT and the time of the mHTT expression (Kim et al., 2016). The proteins, which interact with the oligomers, are important in key cellular functions of RNA-binding, biogenesis, transcription, translation, cytoskeletal organisation, mitochondrial function, and vesicle transport (Ravikumar et al., 2004; Beal, 2005; Kim et al., 2016). The mHTT fibrils and IBs have a different range of interacting proteins than the mHTT oligomers, where IB interact with about 80 proteins (Kim et al., 2016). This is probably due to their more rigid and tightly packed structure and insolubility. The IB are enriched with proteins associated with chaperones and other proteolysis proteins, as the IB show a high concentration of ubiquitin (Tan et al., 2008; Kim et al., 2016; Ramdzan et al., 2017). The proteins interacting with the IB often contain prion-like sequences, which can be easily recruited to the inclusions after their formation (Ramdzan et al., 2017). Electron tomography reveals in artificial systems that IBs also come in contact with intracellular membranes, mostly the endoplasmic reticulum (ER) membrane. The IB and ER membrane interaction leads to the “freezing” of the ER membrane dynamics at the site of interaction. This IB and ER contact results in the reorganisation of the proteins at the membrane and thus impairing the proper function of the ER (Bäuerlein et al., 2017). It is not clear, however, whether interactions of IBs with the ER membrane observed in artificial systems has functional relevance.

In addition to the intracellular effect of mHTT in cells that express it, mHTT is able to spread its aggregating potential to neighbouring cells. The mHTT monomers and aggregates can spread through the late endosomal/lysosomal secretory pathway and via cell-cell contacts through tunnelling nanotubes (Costanzo et al., 2013; Trajkovic et al., 2017), and are also found in the cerebral spinal fluid of patients (Tan et al., 2015). The spread of mHTT leads to the induction of mHTT aggregation in neighbouring cells and leads to the spread of aggregates into other parts of the brain (Babcock and Ganetzky, 2015). Interestingly, the spread of mHTT can also nucleate wtHTT aggregation (Isas et al., 2017). The uptake of mHTT aggregates by healthy cells, not expressing mHTT, increases the percentage of wtHTT dimers compared to monomers, which can lead to wtHTT aggregation (Sameni et al., 2020). Therefore, the spread of mHTT between cells is able to infect healthy cells which do not have the mHTT gene (Jeon et al., 2016) and lead to their functional derailment.

#### 2.1.5 Effect of polyQ Extensions on HTT Turnover

A factor that enhances the cytotoxicity of mHTT is the reduced functionality of the protein quality control and turnover machinery in case of HD. The half life time of wtHTT in Q19 cells is 57 h, while that of mHTT in Q68 cells is reduced to 28 h (Wu et al., 2016). In healthy tissue, protein turnover is controlled by molecular chaperones, the ubiquitin proteasome system (UPS) and lysosomal proteolysis/autophagy systems, such as chaperone-assisted autophagy (CASA) and chaperone-mediated autophagy (CMA). One of the signals enhancing the clearance of wtHTT is its phosphorylation (Thompson et al., 2009). However, in HD, the HTT protein turnover is decreased. The expanded polyQ tract slows down the degradation rate of mHTT, by reducing mHTT phosphorylation (Thompson et al., 2009) and impairing its lysosomal uptake via macroautophagy (Qi et al., 2012). The cellular defence mechanisms attempt to compensate for the reduction in mHTT degradation, by upregulating the CMA pathway. However, over time, the efficiency decreases, which leads to accumulation of mHTT allowing for its aggregation (Koga et al., 2011). Additionally, the UPS system efficiency is reduced, as different components of the system are sequestered by different mHTT aggregate forms (Tan et al., 2008; Kim et al., 2016; Ramdzan et al., 2017). These defects in the protein turnover machinery favour the accumulation of aggregating proteins; all of this only aggravates cellular dysfunction leading to its death.

The current understanding of the molecular basis of HD, allows for the identification of the toxic agent, mHTT, and the different forms of aggregation that it adopts. The awareness of how the different structures induce cell damage leading to cell death provides a promising platform to propose mechanism that could be used to treat the development of HD.

### 2.2 Protein and mRNA Degradation

The gain-of-function of the HTT protein by polyQ extension is critical for the development of HD; therefore, reducing the levels of the mHTT protein may offer a promising therapeutic avenue for HD. In principle, this could be achieved by preventing the production in the first place, or by improving degradation of mHTT aggregates or their precursors. *In vivo* studies indicate that abolishing mHTT expression slows down aggregation formation, thus limiting HD progression and can even reverse the HD associated progressive motor decline (Yamamoto et al., 2000). In addition, reducing the levels of mHTT protein prevents any other possible cytotoxic effect it may have (Bauer et al., 2010). mHTT levels can be reduced by either intercepting in the protein synthesis pathway, through the degradation or blockage of mRNA translation, or by targeting the already existing mHTT monomers or aggregates for degradation. There are a number of biologics-based molecular strategies that can or have the potential to efficiently carry out these functions, using endogenous cellular systems, and target protein aggregation in HD (Table 1).

**TABLE 1:** Molecular strategies to target protein aggregation in HD.

Target molecule	Endogenous system	Method
mHTT mRNA	Pre-mRNA processing	Antisense oligonucleotide (ASO)
	RNA interference (RNAi)	Small interference RNA (siRNA)
		Small hairpin RNA (shRNA)
		Artificial microRNA (artificial miRNA)
mHTT Protein	Ubiquitin proteasome system (UPS)	Proteolysis-targeting chimera (PROTAC)
		Trim-Away
	Autophagy-lysosomal pathway	Autophagosome-tethering compound (ATTEC)
		Autophagy-targeting chimera (AUTEC)
		Lysosome-targeting chimera (LYTAC)
	Chaperone- mediated autophagy (CMA)	CMA adapter molecule

In addition to the biologics-based strategies, small molecules could also be used as potential HD therapeutics. These are small-molecule inhibitors which target either the RNA or the protein and modulate HD levels and aggregation. There are a number of identified inhibitors of mHTT aggregation, *in vitro* and *in vivo* (Pollitt et al., 2003; Zhang et al., 2005; Chopra et al., 2007; Khan et al., 2019; Lo et al., 2020), however their lack of selectivity for the target protein limits them as potential HD therapeutics.

### 2.3 mRNA Based Approach

The mHTT aggregation in HD can be targeted at the post-transcriptional level, through degrading or modulating the translation efficiency of the mHTT mRNA. These approaches prevent mHTT production, reduce its cellular levels and propensity to aggregate, and diminish the mHTT induced cytotoxic effects. The mRNA molecule is easily targetable as it is accessible in the nucleus and the cytoplasm; it is unprotected and lacks repair machinery. This approach of modulating protein production on mRNA level is more feasible than altering gene transcription or the genome itself. Predominantly, mRNA targeting approaches focus on using the antisense nucleotide (ASO) and RNA interference (RNAi) mechanisms.

#### 2.3.1 ASO Based Technologies

**FIGURE 3:**
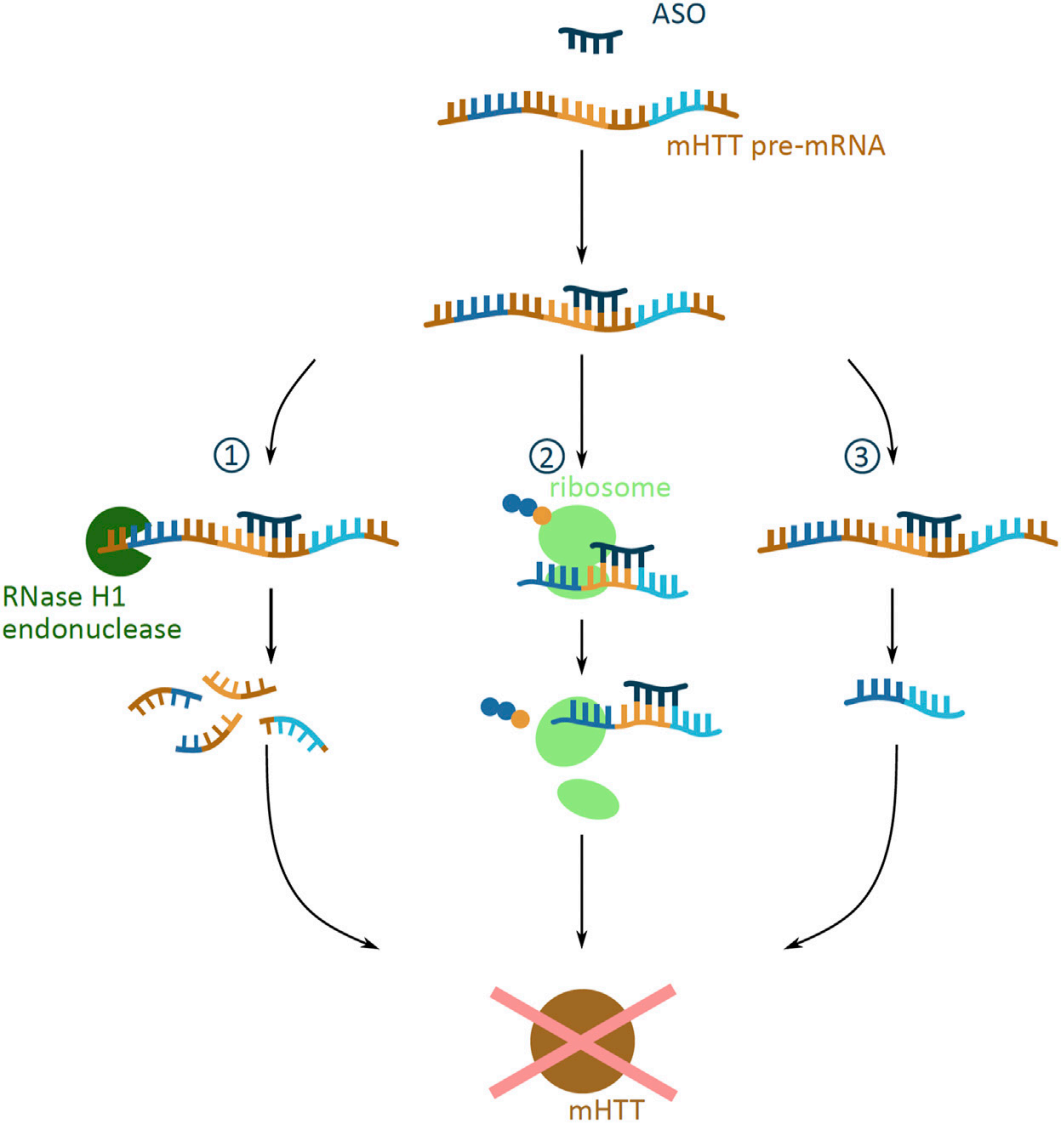
Potential outcomes of antisense oligonucleotide (ASO) approach targeting mHTT mRNA. Schematic presentation of possible outcomes of ASO (dark blue) binding to mHTT pre-mRNA (brown indicates introns; dark blue, yellow and light blue indicate exons), all preventing the formation of the mHTT (brown). The outcomes include **(1)** degradation via the RNase H1 endonuclease (green) mediated degradation; **(2)** translational arrest of the ribosome (light green) on the mHTT mRNA resulting in incomplete translation of mHTT; **(3)** alternative splicing caused by masked splicing sequences, and thus prevention of toxic mHTT expression, but restricted only to an altered non-toxic HTT protein version.

ASOs are short, synthetic, single-stranded oligonucleotide analogues. They are 16–22 nucleotides long and bind predominantly to pre-mRNA through Watson-Crick binding (Southwell et al., 2018). Depending on the site of binding to the mRNA, ASOs can evoke a number of cellular responses which can prevent protein expression (Rinaldi and Wood, 2018) (Figure 3). ASOs binding can cause the degradation of mHTT pre-mRNA by the RNase H1 endonuclease, which recognizes the DNA and RNA duplex (Southwell et al., 2018). Another response is the ASO mediated translational arrest, where the bound oligonucleotide stalls the translational machinery on the mRNA (Gagnon et al., 2010). A third potential outcome is the altering of the pre-mRNA splicing, where ASOs can mask splicing sequences and lead to the expression of an altered and non-toxic protein (Evers et al., 2014).

The design of ASOs can target a number of different sequences that are specific to the mHTT pre-mRNA, allowing for the selective targeting of mHTT production. ASOs can be designed to selectively target the expanded polyQ repeat region of the mHTT mRNA, without affecting the wtHTT mRNA levels (Gagnon et al., 2010). Another ASO target can be one of the 50 identified viable HD-specific SNPs; since ASOs target the pre-mRNA, the presence of both, exons and introns in the sequence increases the number of possible SNP target sites (Carroll et al., 2011). The fine tuning of ASO design to the targeted SNP is vital, as different ASO-SNP combinations demonstrate varied tolerability and efficiency in knocking down protein production (Southwell et al., 2014).

Preclinical tests performed *in vitro* and *in vivo* in mice and non-human primate models suggest that ASOs are able to reduce mHTT in a dose-dependent manner, both in the nucleus and the cytoplasm (Didiot et al., 2018). This ASO-mediated mRNA suppression can reverse the disease course, by rescuing the HD caused motor dysfunction and brain atrophy (Kordasiewicz et al., 2012) and by attenuating the psychiatric and cognitive impairments (Southwell et al., 2018).

The size of the ASO molecules prevent them from passing the blood-brain barrier, therefore ASOs need to be delivered by intrathecal delivery, straight to the cerebrospinal fluid (CSF) (Southwell et al., 2018). Once in the CSF, ASOs efficiently pass through nerve cell membranes, have a long half-life and are effective in low doses (Southwell et al., 2018). Injections into the brain, however, are invasive neurosurgical procedures with considerable risks. Modifications of ASOs increase their metabolic stability and affinity to the pre-mRNA target, greatly enhancing their drug-like properties. These alterations target the phosphate as well as the sugar groups of the molecule (Dhuri et al., 2020). Current advances in ASO-based therapies aim to improve their delivery through the blood-brain barrier by designing new ASO structures, such as the tricyclo-DNA ASO molecules (Imbert et al., 2019), or by encompassing ASOs into nanocarriers (Min et al., 2020).

#### 2.3.2 RNAi Based Technologies

**FIGURE 4:**
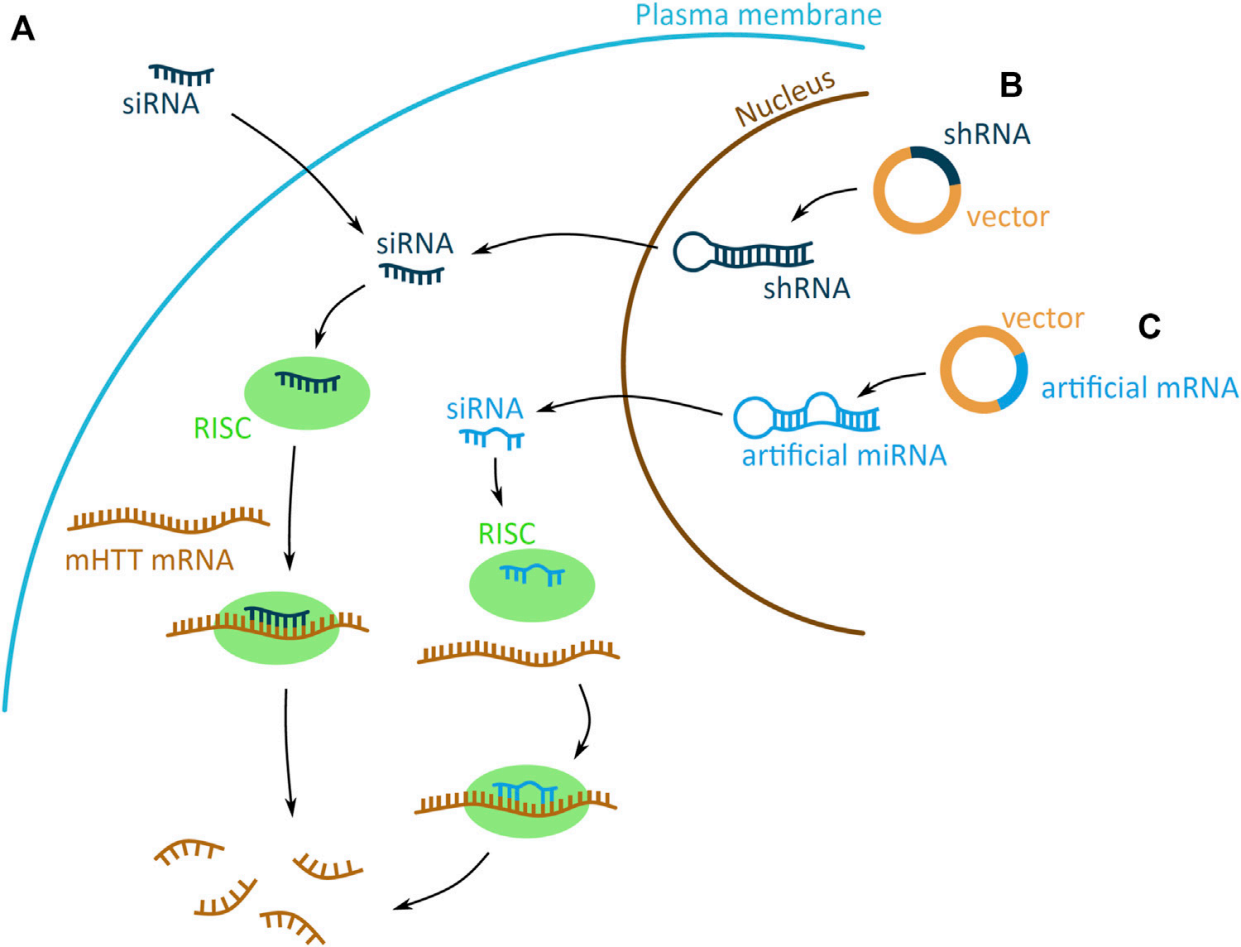
Potential mHTT mRNA targeting approaches using the RNA interference (RNAi) system. The RNAi based technologies use the endogenous system and target mHTT through an artificially introduced siRNA (small interfering) (dark blue) **(A)** or siRNA encoded on a plasmid and expressed in a short hairpin RNA (shRNA) (dark blue) **(B)** or artificial micro RNA (miRNA) (light blue) **(C)** scaffold. **(A)** The artificial siRNA requires no processing and, once inside the cell, binds to the RNA-induced silencing complex (RISC) (green). Driven by the siRNA binding, RISC attaches to mHTT mRNA (brown) and induces mRNA degradation, preventing the expression of mHTT. **(B–C)** The shRNA and artificial miRNA scaffolds enter the cell on a DNA vector (yellow with dark or light blue fragments for shRNA and artificial miRNA, respectively). The shRNA and miRNA are expressed in the nucleus, processed into siRNA and exported into the cytoplasm. There generated siRNA bind to the RISC (green) complex and induces mHTT mRNA (brown) degradation. The shRNA-derived siRNA has a sequence matching perfectly the mRNA, whereas miRNA derived has imperfect matching.

RNAi based technologies can be used as therapeutic approaches to reduce mHTT levels, as they use the endogenous RNAi mechanism that mediates post-transcriptional gene silencing. RNAi is facilitated by a small interfering RNA (siRNA), generated by endoribonuclease processing of a double stranded RNA (dsRNA). The dsRNA is processed to generate the siRNA guide strand, and releases the complementary passenger strand, which is discarded. The siRNA then combines with the RNA-induced silencing complex (RISC), associate with the target mRNA, and induces its degradation (Boudreau et al., 2009). This endogenous RNAi pathway can be highjacked by introducing an artificial siRNA, to target and induce mHTT mRNA degradation. The siRNA molecule can either be delivered into the cell directly as a single stranded siRNA molecule, or encoded on a DNA plasmid and expressed in a dsRNA short hairpin RNA (shRNA) or artificial microRNA (miRNA) scaffold (Figures 4A–C).

#### 2.3.3 siRNA

Introduction of an artificially generated siRNA into the cell is an approach to target mHTT mRNA (Figure 4A). The siRNA needs no further processing, therefore once it enters the cell it can directly induce mHTT mRNA degradation via the RNAi system. Due to the large and negatively charged structure, the siRNA molecule need to be chemically conjugated or modified to enhance its delivery into the nerve cells, prolong its efficacy and reduce its cytotoxicity (Alterman et al., 2019; DiFiglia et al., 2007; Liu et al., 2003). Since, siRNA cannot pass the blood-brain barrier, it needs to be administered by intracerebroventricular injection straight into the CSF, from where it reaches the nerve cells.

The artificial siRNA mediated RNAi approach, first tested in cell culture (Liu et al., 2003), is able to suppress the production of mHTT. *In vivo* studies in animal models, show that siRNA silences mHTT expression, leading to reduced sizes of IB, prolonged longevity of striatal nerve cells, and reduced HD associated motor function disturbances (Alterman et al., 2019; DiFiglia et al., 2007; Wang et al., 2005). Carefully designed siRNA can specifically target mHTT, without disturbing production and function of wtHTT; this specificity is achieved by targeting specific HD-associated SNPs or specific nucleotide inserts or deletions in the sequence (van Bilsen et al., 2008; Pfister et al., 2009; Zhang et al., 2009).

#### 2.3.4 ShRNA

ShRNA scaffold-based expression is preferable to direct siRNA delivery, as it offers a longer-lasting suppression of mHTT, due to the stable expression of siRNA from the vector. Expression of shRNA is under the regulation of an expression promoter, which can induce high levels of shRNA, increasing the mHTT mRNA silencing efficiency (Harper et al., 2005). The siRNA expression from the shRNA scaffold requires the expression and processing of shRNA to generate siRNA, before it can induce mHTT mRNA suppression (Figure 4B). The vector is delivered into the nerve cells via striatal injection of a recombinant adeno-associated virus (rAAV) vector (Rodriguez-Lebron et al., 2005). The vectors allow the successful internalisation and replication of the siRNA encoding DNA vector (Burger et al., 2004).

*In vivo* experiments in animal models on expressing shRNA-encoding siRNA against mHTT demonstrate that shRNA can prevent appearance of aggregates. Tests in mice demonstrate that shRNA expression reduces mHTT mRNA levels and IB formation, leading to improvements in the behavioural phenotype of HD (Harper et al., 2005; Rodriguez-Lebron et al., 2005). Experiments in rats indicate ability of shRNA to prevent the HD induced neurodegeneration of the striatum (Franich et al., 2008) and the possibility of targeting HD specific SNPs, thus increasing the shRNA specificity for mHTT (Drouet et al., 2014).

#### 2.3.5 Artificial miRNA

The use of an artificial miRNA scaffold for siRNA expression is similar to the shRNA-based approach (Figure 4C). Both generate a dsRNA molecule, both require rAAV vector delivery system and Intrastriatal injection, and are able to spread in the brain (Stanek et al., 2014). However, the artificial miRNA shows to be better tolerated in the mouse cerebellum, than the shRNA (Boudreau et al., 2009). Additional modifications of the miRNA scaffold structure can further increase its safety, by reducing unwanted off-target effects that may occur from the discarded passenger strand (Miniarikova et al., 2016).

*In vivo* experiments, which tested the ability and efficiency of artificial miRNA targeting the mHTT mRNA show that rAAV mediated expression of artificial miRNA can transduce over 80% of cells in the mice striatum (Stanek et al., 2014) and can also successfully spread in larger brains, such as that of a minipig (Evers et al., 2018). The expression of miRNA shows to be effective in lowering mHTT in different animal models with ranging brain sizes, from mice, rats, minipigs to sheep (Stanek et al., 2014; Evers et al., 2018; Pfister et al., 2018; Spronck et al., 2019). The lowering of mHTT using artificial miRNA appears to be safe in the putamen of rhesus macaque monkeys (McBride et al., 2011) and other *in vivo* experiments show no major adverse effects of artificial miRNA gene therapy.

#### 2.3.6 State of Research on mRNA Based HD Treatment

A wealth of data indicates the potential of targeting the mHTT mRNA for preventing mHTT protein aggregation and subsequent development of HD. In fact, two molecules targeting mHTT mRNA are in phase I/IIa of clinical trials. First one is ASO IONIS-HTT_Rx_ that shows a dose-dependent ability to reduce mHTT in the CSF of adults with early HD (Tabrizi et al., 2019). Second, is an artificial miRNA molecule, AMT-130, developed by UniQure, for which the first clinical trials begun in 2020 (http://uniqure.com/gene-therapy/huntingtons-disease.php). These two molecules illustrate an unprecedented potential of mRNA targeting approaches to fight HD, even though there is still a long way to being accepted as therapeutics.

Despite promising results in animal experiments, RNA-based therapeutics for HD did not yet hit the clinics. Lack of experience with RNA-based drugs in the human brain means that tolerability and efficiency of a drug can vary between patients and the potential effects on the cellular machinery are not yet fully understood. The most significant limitation is, however, that RNA drugs do not pass the blood-brain barrier. Therapies can circumvent this mainly by two strategies, direct injection into the brain or CSF, or application by adenoviruses. Injections into the brain require a complex neurosurgical procedure. After application, it needs to be ensured that the RNA drug is spread through the tissue. While this is effective in animal models with ranging brain sizes, from mice, rats, minipigs to sheep (Stanek et al., 2014; Evers et al., 2018; Pfister et al., 2018; Spronck et al., 2019), a successful therapy needs to consider the mass of the human brain. The human brain is three orders of magnitude larger than that of the tested animals, making diffusion of a drug throughout the brain more challenging. Additionally, this application would need to be repeated regularly to ensure a constant suppression of the production of mHTT. Application of drugs via an adenovirus may reduce the invasiveness and they may offer long-term effects by installing a stable source for RNA production in nerve cells. However, once administered, the drug cannot be removed from the body and in case of adverse reaction, the drug can become a threat to the patient’s health.

### 2.4 Protein Based Approaches

The protein-based approaches to targeting protein aggregation in HD act through inducing the degradation of the whole mHTT protein, instead of inhibiting its function or physically preventing its aggregation. This approach removes the mutant protein in its different aggregate forms from the cell and inhibits any potentially detrimental effects of a gain-of-function variant. It relates to the disturbance of degradation pathways restoring either UPS or autophagy for aggregation-prone HTT variants. Protein degradation also overcomes potential cellular drug resistance that can be acquired by inhibiting protein function. The removal of proteins is also a fast and reversible method of knocking down of proteins, which allows better temporal control of protein concentration in the cell (Gao et al., 2020). Furthermore, degradation allows for the rapid removal of long-lived proteins, whereas mRNA based methods of preventing protein expression rely on the time of the inherent protein turnover and are not efficient in the degradation of long-lived proteins.

The protein degradation platforms described in this review utilize the endogenous cellular protein degradation mechanisms, namely the UPS or the autophagy-lysosomal pathways. The research targeting protein degradation is in its infancy; nevertheless, it explores promising approaches that could develop into powerful therapeutics.

#### 2.4.1 Ubiquitin Proteasome System

**FIGURE 5:**
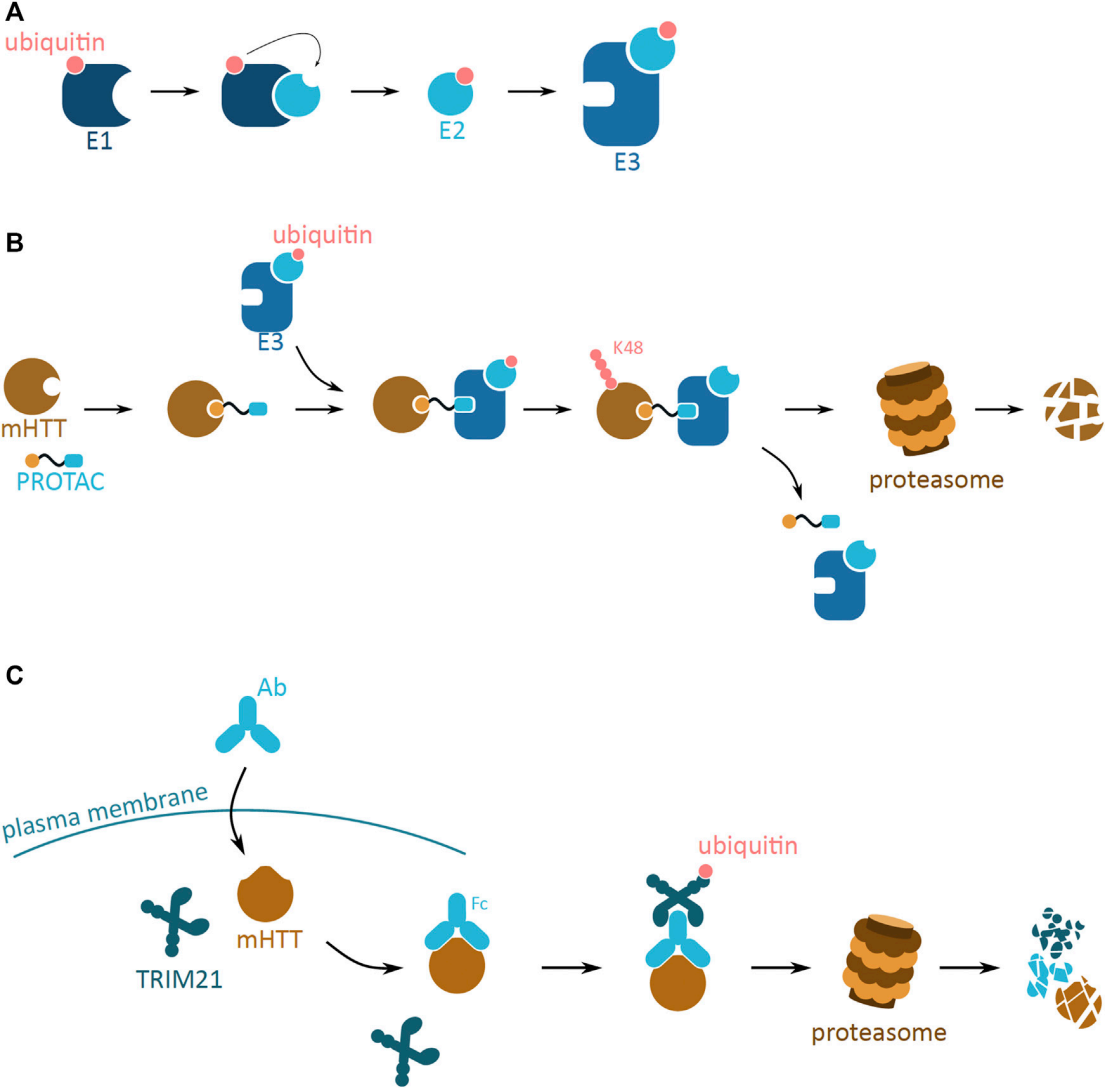
Potential mHTT protein degradation approaches using the ubiquitin proteasome system. **(A)** The ubiquitin activation cascade involving three ligase enzymes, E1, E2, and E3. E1 (dark blue) activates the ubiquitin molecule (pink), which is then transferred to E2 (light blue) conjugating enzyme. Once conjugated to ubiquitin, E2 can bind to E3 ligase enzyme (blue). The E3 ligase is then ready to bind to the target protein to ubiquitinate it. **(B)** Schematic of a proteolysis-targeting chimera (PROTAC) targeting cytoplasmic mHTT for degradation. PROTAC binds to mHTT (brown) via the protein binding domain (yellow). PROTAC then interacts with ready E3 ligase (blue) with activated ubiquitin (pink) via the E3 ligase interacting domain (light blue). Close proximity of E3 ligase induces ubiquitination of mHTT, and process repeats leading to K48 polyubiquitination of mHTT. mHTT is targeted for proteasomal degradation, while PROTAC and E3 detach. **(C)** Schematic of Trim-Away approach targeting cytoplasmic mHTT for degradation. Antibody (light blue; Ab) enters the cell and binds to mHTT (brown). TRIM21 (dark blue) attaches to the Ab and triggers self-ubiquitination (pink). Together the complex undergoes proteasomal degradation.

UPS is one of the principal post-translational modification pathways, responsible for, amongst many, protein quality control, and maintenance of protein homeostasis. One of its main functions is targeting of misfolded proteins to proteasomal degradation. The UPS system relies on the post-translational conjugation of a small regulatory protein, ubiquitin, to the target protein. The ubiquitin tag can be a monomer or a polyubiquitin chain, consecutively attached to the lysine (K) residues of the previous ubiquitin. Depending on the lysine residue attachment, the ubiquitinated protein is destined for different degradation fates—K48 for UPS and K63 for autophagosome. The enzymatic cascade that leads to the covalent binding of ubiquitin molecules to the target protein involves the E1, E2, and E3 ligases which are responsible for activating, conjugating and ligating ubiquitin to the target protein, respectively (Hershko and Ciechanover, 1998) (Figure 5A).

The UPS system has potential to efficiently reduce mHTT aggregation and development of HD, by the means of mHTT degradation. Even though the UPS system is not efficient in endogenously degrading mHTT oligomers and larger aggregates, artificial tagging of mHTT monomers could reduce the mHTT pool and thus slow down aggregation. This approach is employed by two molecular strategies, the proteolysis-targeting chimera (PROTAC) and Trim-Away.

#### 2.4.2 Proteolysis-Targeting Chimera

The proteolysis-targeting chimera (PROTAC) is a small molecule that artificially targets a protein of interest (POI) to the UPS based proteolysis pathway (Sakamoto et al., 2001) (Figure 5B). PROTACs are bifunctional molecules, consisting of a warhead specifically targeting a to-be-degraded protein, and a unit connecting to an E3 ligase. This allows the PROTAC to ensure ubiquitination of the target protein and initiate degradation. The protein targeting domain can be a small molecule, mimicking a known inhibitor of the target protein (Qiu et al., 2021; Yan et al., 2021) or a peptide sequence, designed based on POI crystal structure and the known protein-protein interaction motifs (Jiang et al., 2018; Jin et al., 2020).

PROTAC mediates the interaction between POI and E3 ligase enzyme, leading to the K48 polyubiquitination of POI, targeting it for proteasomal degradation. During this process, PROTAC is not degraded and therefore one molecule can catalyse the ubiquitination of multiple POIs. The PROTAC approach is dependent on UPS; hence, it can only target soluble proteins with cytosolic domains. In the context of mHTT, this approach can target the soluble monomers and oligomers of mHTT. Specifically targeting oligomeric aggregation precursors would allow specific removal of the course of the toxic aggregation without interfering with the biological function of the monomer.

PROTAC molecules efficiently cross the cell membrane and experimental data suggests they can be administered orally or by intraperitoneal injection (Bondeson et al., 2015; Wei et al., 2021). However, due to the size of the PROTAC molecule ranging from 900 to 1,100 Da (Atilaw et al., 2021), it is a challenge to develop PROTACs that may pass through the blood-brain barrier to reach the HD nerve cells, to avoid intrathecal injection into the CSF as discussed for RNA-based drugs. This challenge may become more significant of the PROTAC uses a peptide-based domain as warhead, as this further increases the molecular weight, hence reducing its bioavailability (Jin et al., 2020).

The E3 ligase ligand recruits the E3 ligase to the PROTAC bound POI. Initially, these were peptide based short motifs that endogenously recruit the E3 ligase (Sakamoto et al., 2001; Schneekloth et al., 2004). However, the large size of the peptide renders the PROTAC less efficient in passing through the plasma membrane. The identification of small molecule ligands for a number of E3 ligases, including mouse double minute 2 homologue (MDM2) (Smith et al., 2008), cellular inhibitor of apoptosis protein 1 (cIAP1) (Itoh et al., 2010), cereblon (CRBN) (Winter et al., 2015), and Von Hippel-Lindau (VHL) (Zengerle et al., 2015), allows for the design of more efficient small-molecule PROTACs. Each E3 ligase has a specific range of target proteins; therefore, the possibility of choosing the E3 ligase recruited by PROTAC is crucial for the efficiency of the method (Bondeson et al., 2018). So far, only four E3 ligases are being used in PROTAC, however over 600 E3 ligases are encoded in the human genome, and the ability to use more of them would greatly widen the therapeutic potential of PROTAC to target many more proteins (Bondeson et al., 2018).

Warhead and E3 recruitment unit are connected by a linker. This linker moiety is critical for stability and functionality of the PROTAC. It is often based on alkyl, poly (ethylene glycol) (PEG) or extended glycol chains. These materials are synthetically available, have known flexibilities and their structure can be easily be fine-tuned by robust chemical methods (Troup et al., 2020). The flexibility, length and the attachment site of the linker to the two active domains affects PROTAC potency and efficacy. This is because the correct relative spatial orientation of the POI and the E3 ligase is vital for effective POI ubiquitination (Cyrus et al., 2011; Smith et al., 2019; Sun et al., 2019).

Up to date, over 40 protein targets are successfully degraded using PROTACs (Sun et al., 2019). Most of the targets are cancer related proteins (Qiu et al., 2021; Wei et al., 2021; Yan et al., 2021), with one PROTAC molecule, ARV-110, in a clinical trial (Poh, 2020). The knowledge of PROTAC design and efficiency can be translated into proof-of-concept studies targeting mHTT (Tomoshige et al., 2017, 2018).

The efficiency of mHTT degradation by PROTAC was tested in an *in vitro* cell culture system (Tomoshige et al., 2017, 2018). The PROTACs target mHTT soluble oligomers through small molecule probes, benzothiazole-aniline (BTA) and phenyldiazenyl benzothiazole (PDB), and lead to POI ubiquitination by the cIAP1 E3 ligase. The PROTACs successfully reduce mHTT levels in primary cell cultures derived from HD patients in a dose-dependent manner. In this study, however, the tested PROTACs are unable to differentiate between mHTT and wtHTT, as the levels of the latter are also reduced during treatment (Tomoshige et al., 2017, 2018). This could be a potential downside as the effect that reducing wtHTT levels has on the cell is still unsure. Nevertheless, this preliminary evidence of targeted mHTT degradation provides a promising platform for the development of PROTAC as a HD therapeutic.

#### 2.4.3 Trim-Away

Trim-Away is another method of rapid POI degradation, which utilizes the UPS system (Figure 5C). The approach relies on the antibody based recognition of POI, and binding of the cytoplasmic antibody receptor and simultaneously E3 ubiquitin ligase, TRIM21 (Clift et al., 2017). The TRIM21 E3 ligase is produced in different cell types and tissue, and efficiently attaches to the Fc of the antibody. This binding leads to the autoubiquitination of the TRIM21, and this signal induces proteasome-based degradation of the whole protein complex. Both mHTT and wtHTT proteins have been tested as POIs for the Trim-Away approach (Clift et al., 2017). Microinjection of the designed POI targeting antibody resulted in the selective degradation of mHTT. This method was designed with a purpose of generating protein knockdowns used in protein function research. However, Trim-Away has not yet been successfully applied to develop new therapeutic approaches involving protein degradation.

A drawback of this method as a potential therapeutic is the required delivery of an extracellular antibody into the nerve cells. Methods such as injection, transfection and electroporation are not feasible in a living brain. Due to their large size, antibodies are unable to pass the brain-blood barrier or cell membrane and so require a new functional delivery method that would allow their entry into the brain and the nerve cell.

#### 2.4.4 Autophagy-Lysosomal Pathway

**FIGURE 6:**
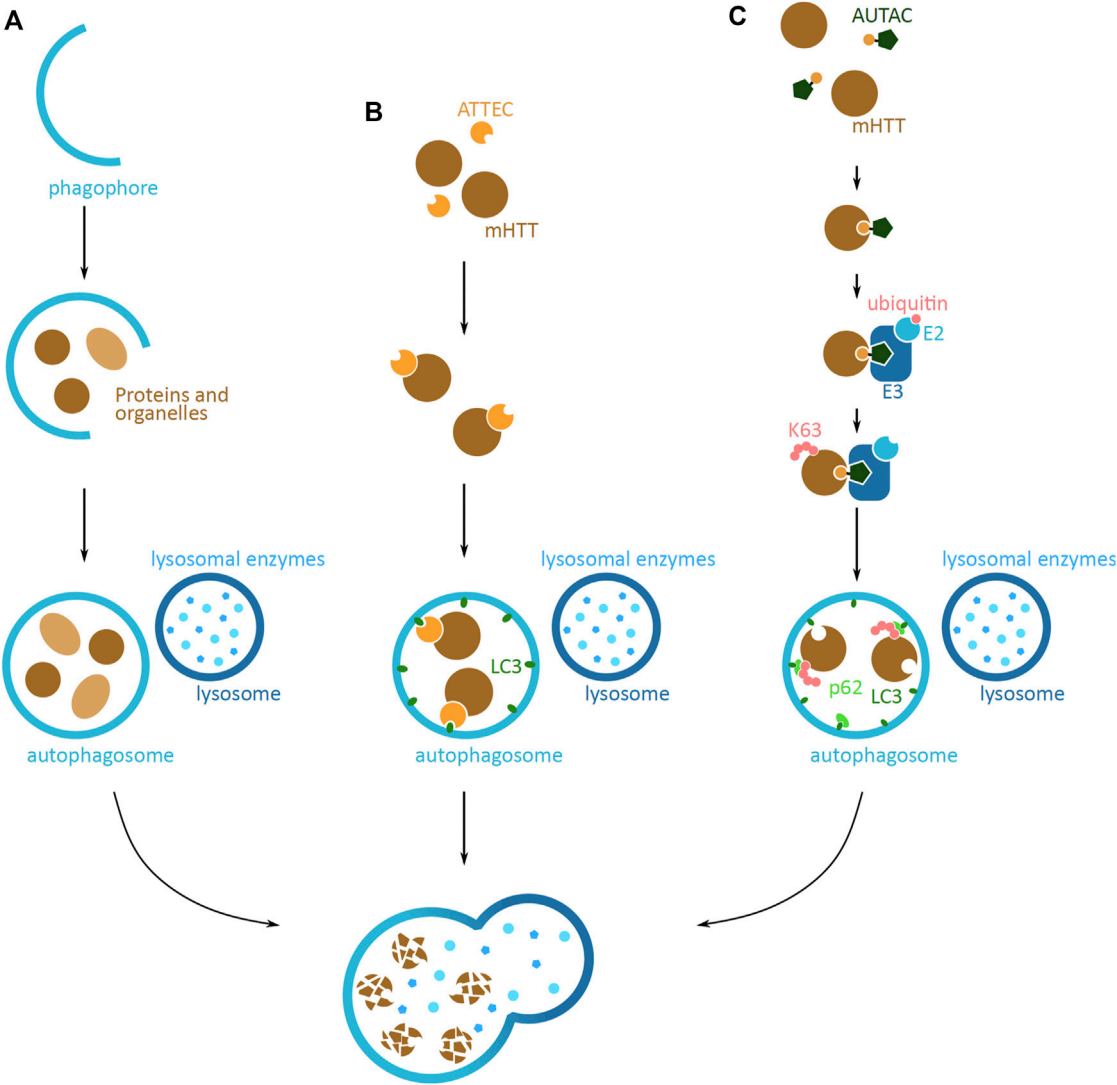
Potential mHTT protein degradation approaches using the autophagy-lysosomal pathway. **(A)** Schematic overview of the autophagy-lysosomal pathway. Lunate-shaped phagophore membrane (blue) forms, and as it elongates it engulfs proteins and organelles (light and dark brown) destined for degradation. The phagophore closes off and becomes an autophagosome vesicle. The autophagosome fuses with the lysosome (dark blue), and the lysosomal enzymes (light blue) degrade the proteins and organelles inside. The same elements are relevant in **(B,C)**, but are not shown in figures. **(B)** Schematic of an autophagosome-tethering compound (ATTEC) targeting cytoplasmic mHTT for degradation. ATTEC (orange) binds to mHTT (brown), and tethers mHTT to LC3 autophagy protein (green). This leads to mHTT being engulfed into the autophagosome (blue). Upon the fusion with the lysosome (dark blue), mHTT is degraded by the lysosomal enzymes (light blue). **(C)** Schematic of an autophagy-targeting chimera (AUTAC) targeting mHTT for degradation. AUTAC attaches to mHTT (brown) via the protein binding domain (yellow). The guanine derivative domain of AUTAC (dark green) is recognized by E3 ubiquitin ligase (dark blue), and close proximity to mHTT, induces mHTT ubiquitination. Multiple rounds of ubiquitination lead to K63 polyubiquitination (pink) of mHTT. The polyubiquitination is recognized by an autophagy reporter p62 (light green), which then tethers mHTT to the phagophore and autophagosome via LC3 (green). Upon fusion with the lysosome (dark blue), mHTT in the phagosome is degraded by the lysosomal enzymes (light blue).

An alternative protein degradation system is the autophagy-lysosomal pathway, which sequesters and breaks down cytoplasmic components via the lysosome (Shaid et al., 2013). Approaches based on this pathway have the potential to target larger mHTT aggregates that cannot be degraded by the UPS system. Endogenously, the autophagy process is initiated by the formation of a phagophore, a lunate-shaped membrane (Figure 6A). The phagophore elongates and engulfs proteins and organelles designated for degradation, finally closing off to form the autophagosome vesicle. The vesicle then fuses with a lysosome, to form the autolysosome, where the lysosomal enzymes degrade the sequestered proteins and organelles. This system is able to degrade proteins in both a selective and non-selective manner, where cellular queues and specific K63 ubiquitin tagging can lead to targeted protein degradation (Shaid et al., 2013).

Autophagy based techniques that operate through the removal of proteins through targeting them to the autophagosome, described below, are the autophagosome-tethering compound (ATTEC) and autophagy-targeting chimera (AUTAC). The method based on the endo-lysosomal degradation pathway is a lysosome-targeting chimera (LYTAC) and another method is using the CMA pathway; in both cases proteins are degraded by targeting them straight to the lysosome.

#### 2.4.5 Autophagosome-Tethering Compound

The autophagosome-tethering compound (ATTEC) is a direct strategy to target POI for degradation via the autophagosome. ATTEC mediates the tethering of POI to LC3, one of the main autophagy proteins found in the phagophore and later autophagosome (Li et al., 2019) (Figure 6B). Once sequestered in the autophagosome, the POI is destined for degradation. A proof-of-concept study identified molecules that efficiently interact with both mHTT and LC3 (Li et al., 2019). The tests conducted in HD patient primary cell lines, *Drosophila* and mouse models with human mHTT, revealed that the molecules can specifically target the mHTT to the autophagosome, leading to mHTT degradation and its reduced levels. The identified molecules do not show any major adverse effect on the cellular function, and interestingly, do not affect the global autophagy function. Additionally, some of the identified molecules can pass through the blood-brain barrier, and can function at low concentrations. Even though the tests so far are based on *in vitro* cell culture models and *in vivo* tests, the preliminary results showing successful and specific mHTT degradation indicate a promising therapeutic potential of ATTEC for HD and other proteopathy diseases (Li et al., 2019).

#### 2.4.6 Autophagy-Targeting Chimera

A recent approach that targets proteins for degradation via autophagosomes, is the autophagy-targeting chimera (AUTAC) based system (Takahashi et al., 2019) (Figure 6C). The principle of targeting proteins is similar to that of ATTEC; however, the structure and function of AUTAC resembles that of PROTAC, as both have a POI targeting domain and a degradation tag, joined by a linker moiety and both induce POI ubiquitination. The AUTAC molecule has a guanine derivate domain, which upon binding to POI mimics a protein post-translational modification of *S-*guanylation. The tag is recognized by the ubiquitination system and leads to the K63 polyubiquitination of the POI. The selective autophagy pathway, via the p62 reporter of autophagy, in turn recognizes this modification and sequesters POI in the autophagosome and destines it for degradation.

The AUTAC approach has not yet been tested on HTT; however, *in vitro* proof-of-concept studies show that this technique can successfully degrade cytosolic proteins as well as larger structures, such as mitochondria. The ability to degrade structures of different sizes makes AUTAC an interesting approach for degrading larger mHTT aggregates. Further *in vitro* and *in vivo* studies, together with the knowledge gained from PROTAC design, can make the AUTAC molecule a very promising HD therapeutic method.

#### 2.4.7 Chaperone-Mediated Autophagy Based Approach

**FIGURE 7:**
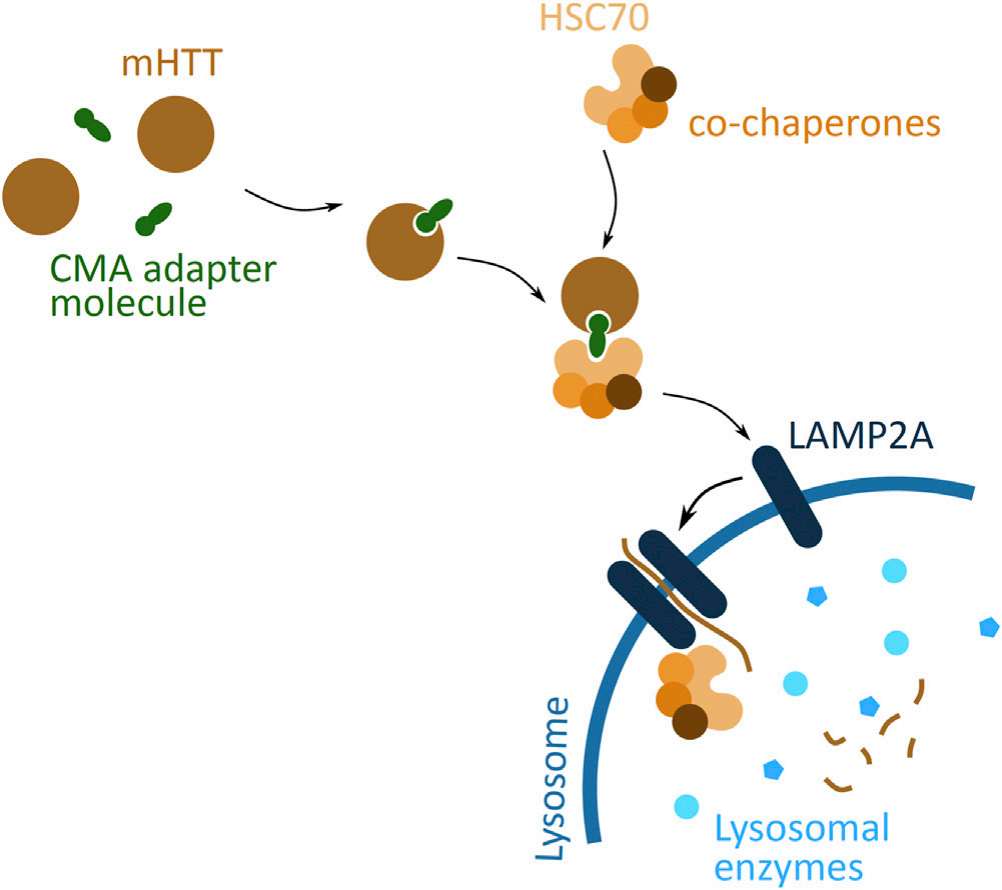
Potential mHTT protein degradation approach using the chaperone-mediated autophagy (CMA) pathway. The CMA adapter molecule (green) binds to cytoplasmic mHTT (brown). The HSC70 chaperone (light orange), together with co-chaperones (darker orange), binds to the adapter molecule. HSC70 interacts with LAMP2A (dark blue), found on the lysosomal membrane (blue), and induces LAMP2A dimerization. The chaperone complex unfolds mHTT protein and together with mHTT passes through the LAMP2A formed channel into the lysosome, where all is degraded by the lysosomal enzymes (light blue).

A different cellular pathway, based on lysosomal degradation, considered for targeted protein degradation is CMA (Figure 7). CMA targeted degradation is based on the chaperone-dependent selection of protein targeted for degradation, and its direct translocation across the lysosomal membrane. An adapter molecule facilitates the interaction between POI and the chaperone complex, HSC70 and co-chaperones. The translocation through the lysosome membrane occurs through interaction of HSC70 with a transmembrane lysosomal protein, lysosome-associated membrane protein 2 (LAMP2A). This binding induces LAMP2A multimerisation and simultaneously the chaperone complex unfolds the POI. The POI then passes through the formed LAMP2A channel, and into the lysosome, where it is degraded (Xilouri and Stefanis, 2015).

A proof-of-concept study has demonstrated feasibility of the CMA based approach of targeted mHTT degradation (Bauer et al., 2010). The molecule used to induce mHTT degradation, called RHQ, had a peptide sequence that interacts with the polyQ tract, and a HSC70 binding motif. *In vitro* studies show that RHQ is able to distinguish between the polyQ size difference between mHTT and wtHTT, and selectively induce mHTT monomer degradation. The *in vivo* experiments indicate the efficiency of RHQ in degrading mHTT in.

The mice striatum and reversing the HD symptoms, by improving metabolic and neurobehavioral outcomes. A shortcoming of this approach is the peptide structure of the molecule, which reduces the efficiency of passing through the plasma membrane, and requires its delivery *in vivo* via the rAAV approach. Nevertheless, the CMA based approach presents yet another potential therapeutic solution for targeting protein aggregation in HD, by targeting mHTT to the lysosome.

#### 2.4.8 Lysosome-Targeting Chimera

**FIGURE 8:**
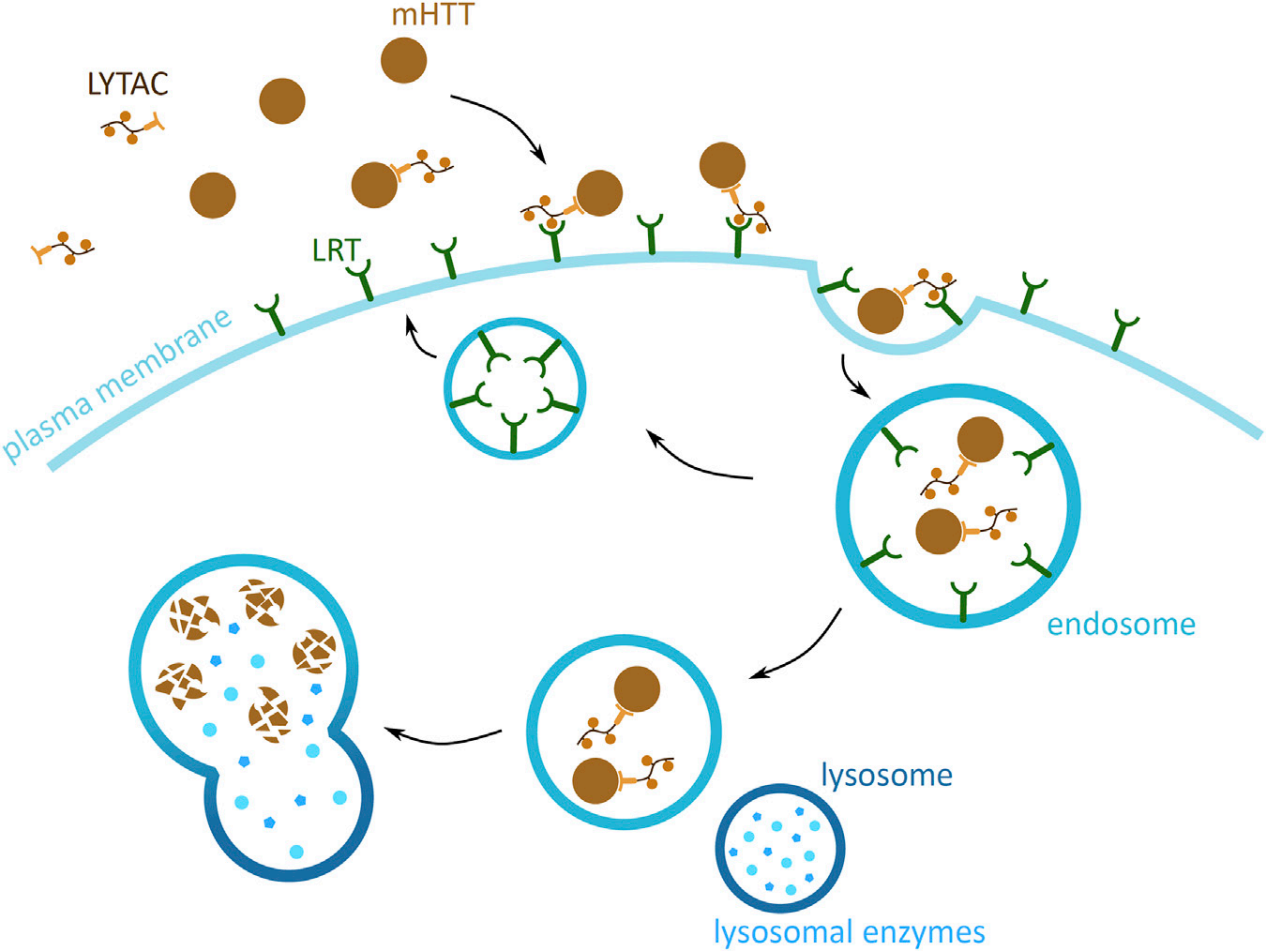
Potential mHTT protein degradation approach using the endosomal/lysosomal pathway. Schematic presentation of a lysosome-targeting chimera (LYTAC) targeting extracellular mHTT for degradation. LYTAC binds to mHTT via the protein-binding domain (light orange) and then interacts with lysosome targeting receptors (LTR) (green) via the LTR motif domain (orange). Subsequently, mHTT bound to LTR via LYTAC is then internalised into the cell, through an endosome (blue). mHTT with LYTAC is then released form LTR, and LTR are recycled back to the plasma membrane. mHTT is degraded by the lysosomal enzymes (light blue), and upon endosome and lysosome (dark blue) fusion.

The previously presented methods focus on the degradation of cytoplasmic mHTT monomers and aggregates. However, mHTT is also found in the extracellular space, where it induces the spread of mHTT aggregation in the brain tissue. This form of cytotoxic mHTT can potentially be targeted using the lysosome-targeting chimera (LYTAC) technology, which exploits the endosomal/lysosomal pathway and triggers the degradation of extracellular and membrane bound proteins (Banik et al., 2020) (Figure 8). The approach is based on tethering the POI to a lysosome targeting receptor (LTR) via the LYTAC molecule. The POI attaches to LYTAC by a targeting sequence, which is either an antibody (Ahn et al., 2021; Banik et al., 2020) or a small synthetic peptide (Ahn et al., 2021). This interaction of POI and LTR induces the internalisation of the complex by endocytosis. Upon the change in pH of the endosome, the POI and LYTAC is released from the LTR, which is then recycled back to the membrane. The POI and LYTAC are degraded by proteases, after the endosome and lysosome fusion.

There are no studies of applying this method to degrade mHTT, but two proof-of-concept studies show that this method successfully degrades both, membrane bound and extracellular proteins, both *in vitro* and *in vivo* (Ahn et al., 2021; Banik et al., 2020). Additionally, the targeting of POI has been shown in both, a tissue-specific and non-specific manner. This can be achieved by the specialised targeting of a LTR by the LYTAC LTR targeting motif (Ahn et al., 2021; Banik et al., 2020). Together, these features of LYTAC demonstrate unprecedented potential for targeting extracellular mHTT in the brain and the central nervous system. Being able to degrade free flowing mHTT can reduce its potential of infecting new cells and nucleating mHTT and wtHTT aggregation, thus slowing down the spread of HD in the brain.

### 2.5 Modulating Protein Quality Control

An important factor in preventing HTT aggregation is the cellular protein quality control machinery, and it is an intriguing thought to exploit this system to complement other therapeutic approaches. Chaperones interact with HTT, specifically the Hsp70 machinery consisting of HSC70, the nucleotide exchange factor HSP110 and the J-domain protein DNAJB1 (Monsellier et al., 2015; Scior et al., 2018). Loss of a functional Hsp70 system increases aggregation of mHTT (Scior et al., 2018; Rodríguez-González et al., 2020). *In vitro* and *in vivo* studies show that this chaperone machine can supress amyloid fibril formation, reduce aggregation and also lead to disaggregation of already formed amyloid fibrils (Monsellier et al., 2015; Kakkar et al., 2016; Scior et al., 2018). Interestingly, lowering the chaperone levels can also have a protective function, by reducing the diassagregation of aggregates and therefore the spread of nucleating agents (Tittelmeier et al., 2020). This method of reducing HTT aggregation depends on the precise regulation of chaperone activity in the HD affected cells. This regulation could be modulated by pharmacological agents, for example by Hsp90 inhibitors such as geldanamycin, which induce chaperone production (Roe et al., 1999; Fujikake et al., 2008). Another approach of modifying chaperone activity could be post-translational modifications introduced to the chaperone protein, which enhance their anti-aggregation activity (Lindstedt et al., 2019). The drawbacks of this method as a therapeutic against HD are the difficulty of treatment delivery and lack of its specificity only to the HD affected cells, and the potential adverse effects that altering of the chaperone production and function could have on the cellular pathways.

## 3 Future Direction

There is a wealth of research on the potential molecular methods to target protein aggregation in HD. HD is caused by a trinucleotide expansion mutation in the HTT protein, which generates a mutant protein prone to aggregation. The formation of different mHTT aggregate structures induces a highly cytotoxic effect, leading to nerve cell death, brain atrophy and neurodegeneration. Accumulating experimental evidence demonstrates that lowering mHTT levels ameliorates HD symptoms and disease development. The proof-of-concept and preclinical studies show the possibility or potential of molecular methods to target mHTT and reduce its levels in the cell. The different mRNA and protein targeting techniques present a promising avenue for finding a treatment for HD by removing mHTT oligomers.

Development of drugs targeting HD could profit from progress on targeting protein aggregation in other neurodegenerative diseases, such as Alzheimer’s disease and Parkinson’s disease. Successful development of an effective treatment of mHTT aggregation could then be expanded to develop treatment for other protein aggregation diseases caused by polyQ expansion (Evers et al., 2011; Yamashita et al., 2019) or by other proteins, such as in Alzheimer’s and Parkinson’s diseases. However, before the potential treatment methods enter clinical trials, a number of issues need to be resolved regarding both, the target and the targeting molecule.

Several challenges also emerge with the fact that the targeting of mHTT protein production and aggregation is done in the nerve cells. Firstly, it remains to be elucidated whether the soluble aggregates or the fibrils are the HD causative agents, making the targeting of the toxic molecule more complicated. Secondly, the nerve cells targeted for treatment do not undergo further mitosis, meaning any manipulation leading to cell death leads to permanent cell loss. Thirdly, the interference in protein production and aggregation needs to be selective and to precisely target degradation of the cytotoxic agent, not to disturb other endogenous pathways, cause adverse side effects and lead to fatal loss of neurons.

### 3.1 The Target Molecule

The well-understood molecular basis of HD is a good base for the development and preliminary design of possible treatments. However, several knowledge gaps remain and progress in addressing them would facilitate finding effective therapeutic methods for treating HD. One them is identification of the exact molecular agent that is toxic and causes most of the damage in the nerve cell. Both, the soluble aggregates and insoluble fibrils interact with a wide range of proteins, but it is still debatable which one is more toxic. Some studies suggest that the soluble aggregates are more dangerous for the cell and the fibrils are a safety mechanism to sequester the aggregates (Kim et al., 2016; Xi et al., 2016; Ramdzan et al., 2017), whereas others claim that the fibrils are most cytotoxic (Drombosky et al., 2018). Precise identification of the toxic agent would enable a redirection of efforts and more focused development of targeting techniques.

Additionally, a more in-depth understanding of the structure of different mHTT aggregate forms could advance identification of sites that can be used for targeting the protein and aggregates for degradation. For now, the HTT monomer structure has been resolved by Cryo-EM (Guo et al., 2018), and *in vitro* studies allowed a better understanding of the fibril structure and shapes (Bäuerlein et al., 2017; Boatz et al., 2020). However, the *in vivo* structures of the oligomers, soluble aggregates, and fibrils, are not well understood. The identification of aggregate specific structures and targetable protein domains, for example the N- and C-terminal domains, would allow for more precise targeting of the toxic mHTT, while protecting the wtHTT.

Better insight into the function of wtHTT in the organism would allow for improved assessment of the potential effect of its removal. The targeted reduction of mHTT leads to an overall reduced HTT levels in the cell; therefore, it would be important to understand potential effects of lowering of HTT on the cell. Some studies indicate it to have little effect on the cells or organisms studied (McBride et al., 2011; Stanek et al., 2014; Tomoshige et al., 2017; Alterman et al., 2019; Tabrizi et al., 2019), whereas others indicate a potentially detrimental effect of long term wtHTT loss on the brain (Caron et al., 2020). This is vital, as research on the mHTT reducing methods aims to be translated in treatment in humans. Additionally, a better understanding of the quality control of mHTT and how its derailment can lead to mHTT aggregation, can open new avenues of research. Techniques that focus on these mechanisms could ensure prolonged and functional mHTT degradation, preventing aggregate formation and HD development.

### 3.2 The Targeting Molecule

The presented molecular strategies to target mHTT aggregation in HD are promising and the experimental research shows their potential, by inducing the degradation of mHTT at the pre- and post-translational level. However, a number of crucial concerns remain before these molecular strategies are translated to human clinical trials.

One such concern is that most molecular strategies, as their efficiencies are based on proof-of-concept trials in cell culture or model organisms with much smaller brains than human, such as mice. Despite similarities at cellular level, the orders of magnitude larger size of the human brain limits comparability for transport processes through the brain. This is an issue for both mRNA targeting methods and protein-based approaches. Even though the methods were tested for a wide range of animals, including mice, rats, minipigs, and sheep and rhesus macaques, the dimensions of the human brain are much more extensive than that of animals. Size and structure of brains of different organisms can affect the molecule’s pharmacokinetics and pharmacodynamics, affecting the way it carries out its function. Thus, a compound working in one organism may not have sufficient activity in another, and this is a key challenge for drug delivery.

Another challenge is the importance to exclude side effects. Neurons do not undergo further mitosis. As these cells do not regenerate, possible side effects could lead to irreversible damage. Some of the mRNA and protein targeting approaches show the inability to selectively target the mHTT molecule from the HTT protein pool, and also reduce wtHTT levels. Selectivity is critical to reduce any potential negative effects that reducing wtHTT availability might have on the cell. Additionally, off-target effects may influence other functions of the cell, for instance by overwhelming the endogenous systems leading to the loss of their efficacy. Overexpression of shRNA targeting the mHTT mRNA can interfere with endogenous RNAi processing (Grimm et al., 2006; Boudreau et al., 2009). Addressing such possible off-target effects is vital for any therapeutic strategy in neurons.

Considering advantages and disadvantages of different molecular strategies against mHTT aggregation, a picture emerges which traits an ideal therapeutic against HD may have. These traits include high selectivity of the drug, which would target specifically the mutant protein, in either monomeric or oligomeric form, to prevent any further aggregation and fibril formation at the earliest stage. The therapeutic would also be able to pass through the blood-brain barrier allowing for an easier and less invasive form of administration of the drug to the patient. Ideally, the drug would be able to diffuse easily through the whole brain tissue preventing the spread of the disease to other brain parts. Finally, the molecular strategy should not interfere with any other cellular mechanisms, to ensure no side effects and potential damage to the cell.

## 4 Conclusion

This review is presenting the wealth of research conducted, with the aim of finding treatment for a protein aggregation based disease, HD. The examined approaches are in different stages of preclinical and proof-of-concept experiments. In-depth understanding of the molecular process of mHTT aggregation, the targeting chemistry and bioavailability of the drug are needed to boost translation from preclinical studies to treatments in humans. The new strategies presented here are promising for targeting a protein such as HTT with no known inhibition sites. Progress made in different experiments on treating polyQ and aggregating protein diseases, such as HD, can be also beneficial for developing treatments for other aggregate diseases, such as Alzheimer’s, spinocerebellar ataxia types and Parkinson’s disease, which are detrimental for affected individuals and have a huge societal impact. Compared to these diseases, development of a treatment strategy for HD has a much clearer focus: It is known that the aggregation process of Htt is causing the disease, and any drug must enter the nerve cells. This is a tremendous challenge for developing biologics towards clinical application. This challenge is also a chance—we know who the villain is and where to send the police to round them up.

## Glossary

artificial miRNA: artificial micro RNA
ASO: antisense oligonucleotide
ATTEC: autophagosome-tethering compound
AUTAC: autophagy-targeting chimera
BTA: benzothiazole-aniline
CASA: chaperone-assisted autophagy
cIAP1: cellular inhibitor of apoptosis protein 1
CMA: chaperone-mediated autophagy
CRBN: cereblon
CSF: cerebrospinal fluid
dsRNA: double stranded RNA
ER: endoplasmic reticulum
HD: Huntington’s disease
HEAT: Huntingtin, Elongation factor 3, protein phosphatase 2A, the yeast kinase TOR1
HTT: huntingtin
**HTT** ^**NT**^: N-terminal region
IB: inclusion bodies
K: lysine
LAMP2A: lysosome-associated membrane protein 2
LTR: lysosome targeting receptor
LYTAC: lysosome-targeting chimera
MDM2: mouse double minute 2 homologue
mHTT: mutant HTT
PDB: phenyldiazenyl benzothiazole
PEG: poly(ethylene glycol
POI: protein of interest
polyQ: polyglutamine
PRD: proline-rich domain
PROTAC: proteolysis-targeting chimera
rAAV: adeno-associated virus
RISC: RNA-induced silencing complex
RNAi: RNA interference
shRNA: small hairpin RNA
siRNA: small interference RNA siRNA
SNPs: single nucleotide polymorphisims
UPS: ubiquitin proteasome system
VHL: Von Hippel-Lindau
wtHTT: wild-type HTT.
